# From Genes to Treatment: Literature Review and Perspectives on Acid Sphingomyelinase Deficiency in Children

**DOI:** 10.3390/diagnostics15070804

**Published:** 2025-03-21

**Authors:** Raluca Maria Vlad, Ruxandra Dobritoiu, Daniela Pacurar

**Affiliations:** 1 Department of Paediatrics, “Carol Davila” University of Medicine and Pharmacy, 020021 Bucharest, Romania; raluca.vlad@umfcd.ro (R.M.V.); daniela.pacurar@umfcd.ro (D.P.); 2 “Grigore Alexandrescu” Emergency Children’s Hospital, Bld. Iancu de Hunedoara 30-32 Bucharest, 011743 Bucharest, Romania

**Keywords:** Niemann–Pick, acid sphingomyelinase deficiency, metabolic disorder, sphingomyelin, enzyme replacement therapy

## Abstract

**Background:** Acid sphingomyelinase deficiency (ASMD), most commonly known as Niemann–Pick disease (NPD), is a rare progressive genetic disorder regarding lipid storage. Subtypes A and B are inherited in an autosomal recessive fashion and consist of a genetic defect which affects the sphingomyelin phosphodiesterase 1 gene, leading to residual or lack of enzymatic activity of acid sphingomyelinase (ASM). **Materials and Methods:** This paper provides a brief history and overview to date of the disease and a comprehensive review of the current literature on ASMD in children, conducted on published papers from the past 10 years. **Results:** We identified 19 original publications (16 individual case reports and three series of cases—30 patients). The male/female ratio was 1.4. The youngest patient at disease onset was a female newborn with NPD-A. The youngest patient was diagnosed at 4 months. The longest timeframe between onset symptoms and diagnostic moment was 5 years 3 months. A total of nine patients exhibited red cherry macular spots. A total of 13 children exhibited associated lung disease, and four NPD-A patients with pulmonary disease died due to respiratory complications. A total of 11 children exhibited associated growth impairment. Genetic assays were performed in 25 cases (15 homozygous; 9 heterozygous). A total of four children (13.3%) received enzyme replacement therapy (ERT). Therapy outcomes included decreased liver and spleen volumes, improved platelet and leukocytes counts, and body mass index and stature improvement. **Conclusions:** Sometimes, a small child with a big belly hides a huge dilemma; inherited metabolic disorders are here to challenge clinicians and set the record straight, and genetics is the way of the future in terms of diagnosis and novel treatments. NPD must be considered children with persistent and progressive hepatosplenomegaly and growth failure. Diagnosis requires good clinical skills and access to genetic assays. Since 2022, the FDA has given a green light to a revolutionary enzymatic replacement therapy with human recombinant ASM called Olipudase-alfa. Clinical trial outcomes support its reliability and efficacy in the pediatric population.

## 1. History and Overview of Acid Sphingomyelinase Deficiency

Lysosomal storage disorders (LSDs) are a rare group of heterogenous genetic disorders with multisystem involvement. More than 50 LSDs have been described. Ages of onset vary from the perinatal period to adulthood, and the spectrum of clinical features depends on the most affected organ or system. These diseases most commonly cause liver damage, whether it is asymptomatic hepatomegaly or advance cirrhosis. Splenomegaly, coarse facial features, and neurological impairment accompany the clinical picture of LSD. The combined prevalence of LSD is 1 to 7000 live births, which might come across as a high number, but keep in mind that these pathologies are seen as scarce among the general population [1,2,3,4,5].

Lysosomes are intracellular organelles considered recycling centers, where large, potentially harmful substances are broken-down to be reused by the body. They undertake a vital job: catabolism of a wide range of macromolecules. Each of these are bound to a particular molecule and are essential for its degradation. When enzymes are missing or there is a defect in their activity, specific materials will accumulate in the lysosomes, leading to cellular impairment [3,4,5].

**Figure 1:**
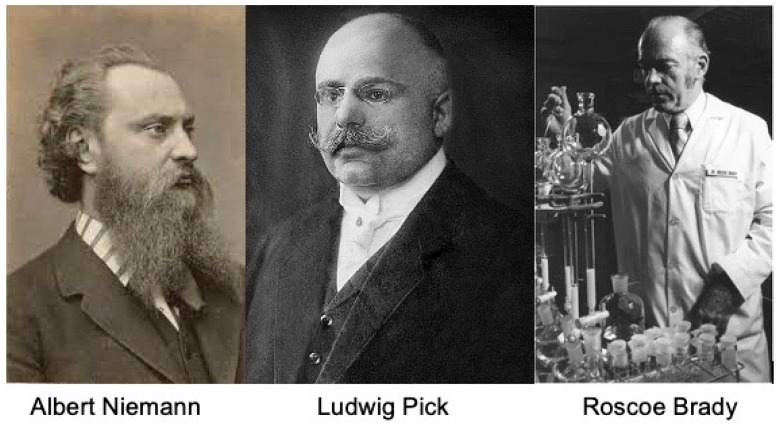
Illustrious figures in the history of Niemann–Pick disease [6].

To understand a concept, weather is a political idea, an engine car, or a groundbreaking treatment, one must dig into the past. Figure 1 displays the “founding fathers” of Niemann–Pick Disease, each playing an essential role in describing and explaining the disease.

The history of Niemann–Pick disease goes way back into the 20th century, particularly to 1914, when German pediatrician Albert Niemann described a young child with jaundice, hepatosplenomegaly, and nervous system impairment, completing a report of “Irene D”. The patient died at 18 months of age. At autopsy, fatty large vacuolated cells had replaced the normal liver and spleen structure. Doctor Niemann believed these cells were fairly similar to those found in Gaucher disease [6,7].

Later, in 1926, German pathologist Dr. Ludwig Pick accurately described the pathology of NPD in a series of papers. He identified the “Pick cell”, a type of foamy cell found in the spleen and bone marrow of NPD patients. In 1934, Klenk identified the lipids accumulating in NPD as sphingomyelins (sphingosine, choline, a fatty acid, and a phosphoric acid). That was an early suggestion that this disease was due to the lack of an enzyme that catalyzed degradation of sphingomyelin. So, it was an entirely different lipid storage disease [6,7].

In 1961, Allan Crocker and Sidney Farber divided NPD into four subcategories (A→D), depending on the patient’s phenotype, the specific symptoms expressed, and the presence of unique “foam cells”. In 1965, Roscoe Brady demonstrated a profound decrease in acid sphingomyelinase activity in affected cells (from rat liver) for NPD types A and B, but not for types C and D. In 1966, Dr. Brady and his co-workers described a sphingomyelin-cleaving enzyme from rat liver. After that, they identified this enzyme deficiency in tissue samples obtained from infants with NPD parents. One year later, Brady characterized acid sphyngomyelinase activity in peripheral blood leukocytes, thus defining NPD-A and NPD-B types. But the journey went on, and Dr. Brady found another group of patients with similar features to ASMD types A and B, but who associated severe neurological impairment; he distinguished this group as NPD type C. In the late 1980s, an investigator identified a defect in the cholesterol metabolism of NPD-C patients, clearly discriminating them from types A and B. The first reliable diagnostic tests applicable to cultured skin fibroblasts for ASMD types A and B [6,7] were brought forward in 1988.

The quest for a cure kept going. In 2022, an exogenous source of ASM to prevent lysosomal accumulation of sphingomyelin in body tissues, called Olipudase-alfa, was put on the market. This molecule was developed for the treatment of non-neurological ASMD and was first approved by the FDA in August 2022. Since then, it has proved its efficacy and safety, both in pediatric and adult populations [8,9,10,11].

Acid sphingomyelinase deficiency (ASMD) is a rare lysosomal storage disorder. Subtypes A and B, most commonly known as Niemann–Pick disease type A (NPD-A) and type B (NPD-B) are rare genetically inherited conditions, with a prevalence between 0.4 and 0.6 to 100.000 live births. The incidence of NPD-A is 1 in 250,000 in the general population, but higher in Ashkenazi Jews (1 in 40,000) [1,2,4,5,12,13]. Niemann–Pick disease type C is a completely different genetic disease, the metabolic defect resulting in cholesterol esterification issues, and this condition is not the subject of this paper.

**Figure 2:**
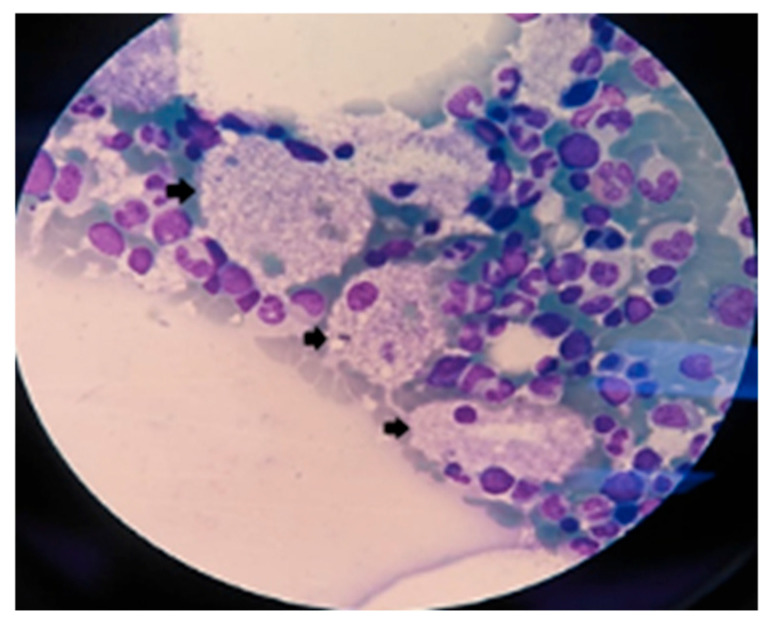
Bone marrow biopsy in ASMD—arrows show “foam cells” [18].

Both A and B types consist of a genetic defect which affects the sphingomyelin phosphodiesterase 1 (SMPD1) gene, leading to residual or lack of enzymatic activity, and so lysosomes are unable to break-down a fat called sphingomyelin. This fat is included in the membrane of many different cells. When body cells get old, they are eaten by macrophages. The immune system cells contain lysosomes, which crack-down sphingomyelin by using an enzyme called acid sphingomyelinase [1,2,3,4,5]. Mutations in the SMPD1 gene lead to complete absence of sphingomyelinase activity, as in NPD-A, while NPD-B has some residual enzymatic activity remaining (5–20%) [14,15,16,17]. In this way, sphingomyelin primarily accumulates in lysosomes of macrophages, cells that travel throughout the body and cause damage in multiple organs and tissues. Macrophages develop a specific lipid-laden microscopic appearance, becoming the histological hallmark of this pathology; they are called “Niemann–Pick cells” or “foam cells” (Figure 2) [1,2,4,18,19].

NPD-A onset is in early infancy, and the disease course is marked by a rapid progression to systemic manifestations and neurological degeneration. These children rarely make it over 3 years of age, death usually occurring from extrahepatic involvement (terminal liver disease is not common in type A). On the other hand, NPD-B patients are considered to be “favored”; chronic visceral ASMD has a slow, progressive course, mainly affecting the liver, with a variable onset from infancy to adulthood, and a lack of neurological symptoms. Most patients survive into adulthood. Death in visceral ASMD occurs later in life from recurrent respiratory infections, advanced cirrhosis, massive hemorrhage by spleen rupture, or neuronal degeneration. In addition, there are patients who fall somewhere between type A and B [1,4,5,20]. Chronic neurovisceral ASM deficiency is an intermediate NPD-A/NPD-B phenotype defined by later onset and slowed neurological/visceral disease progression, also known as NPD type A/B (NPD-A/B) [21,22]. The main features of NPD-A and -B are shown in Table 1 [1,4,5,20].

A differential diagnosis of ASMD types A and B should include Gaucher disease and Niemann–Pick Disease type C. Patients with type 1 non-neuronopathic Gaucher disease present with splenomegaly, anemia, and low platelet numbers, which leads to nosebleeds or easily induced bruises. They may also exhibit enlarged liver, bone pain, or pathologic fractures. Type 2 Gaucher disease debuts at birth or in early infancy, and alongside organomegaly, it presents with seizures and increased muscle tone. Children with chronic neuronopathic type 3 Gaucher disease have progressive neurological impairment from infancy. Diagnosis of Gaucher disease is confirmed through measurement of glucocerebrosidase activity in leukocytes and analysis of the GBA1 gene [23,24]. NPD type C patients are unable to transport cholesterol and other fatty substances inside cells, so lipids accumulate in various body tissues, including the brain. Onset of NPD-C is highly variable, from the neonatal period to isolated cases diagnosed in late adulthood. Neurological impairment is a constant sign of the disease. Diagnosis is confirmed by molecular gene sequencing of NPC1 and NPC2 genes [25,26,27].

Because NPD types A and B are inherited in an autosomal recessive fashion, genetic counselling is crucial for future parents. Keeping in mind the Mendelian pattern, if both parents are known to be heterozygous for a pathogenic SMPD1 variant, they will have a 25% chance of conceiving an affected child. We must also remember that ERT has not been studied in pregnant women. Thus, affected individuals must clearly know their genetic status before thinking of having off-spring [8,10,11,27].

In terms of diagnostic methods, ASM deficiency requires a high suspicion index due to its heterogenic phenotype. Clinical examinations showing liver and spleen enlargement, laboratory investigations displaying low white blood cell numbers and thrombocytopenia, low/absent enzymatic activity of ASM in cell or tissue extracts, imaging investigations showing alterations in liver/spleen/lungs/brain, and eye examinations describing red-cherry spots on the macula must lead to one diagnosis: Niemann–Pick disease [28,29,30,31].

Subtypes A and B are distinguished through laboratory assessment, by measuring low ASM activity and elevated levels of the biomarker lyso-sphingomyelin, which is also used to monitor efficacy of treatment [10,29]. But heterozygote detection is not reliable by enzyme assay and requires molecular study [17,32]. In other words, genetic testing for pathogenic mutations is the gold-standard diagnostic method for ASMD, even more so with the full-length cDNA and genomic sequence encoding ASM being isolated and defined [15,16,17]. Prenatal diagnosis is also available through enzymatic or molecular assay from amniocytes and chorionic villi [17,33].

The liver volume should be obtained every 3 to 6 months in order to assess disease progression. Liver morphology exhibits vacuoles in Kupffer cells because of sphingomyelin and cholesterol build-ups, which react strongly positive with Sudan black B and oil red O but negatively or weakly positive with PAS stain. Electron-opaque, concentrically laminated inclusions within the macrophage cytoplasm are observed using electronic microscopy [1,4,32,34]. Liver fibrosis remains a hallmark of NPD, so fibro-scan measurements are required for clear assessment of these patients [4,12,28]. Spleen assessment includes routine volume measurements from imaging studies and platelet counts because even in NPD-B patients with the mild-kind, slow-progressive disease, there have been reported early sudden deaths after massive hemorrhages from spleen ruptures [12,28,35].

**Figure 3:**
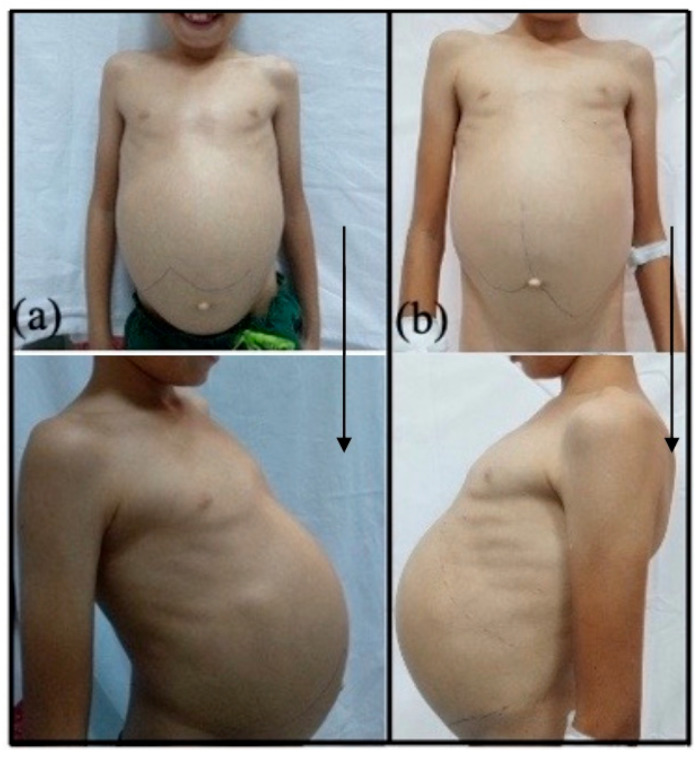
Clinical evolution of organomegaly in a NPD-A/B male. (**a**) At age 9 (BMI = 12.1 kg/m^2^; −3.44 SD), the patient had an abdominal circumference of 70 cm, lower liver edge 10 cm under costal rim, and lower spleen edge 9 cm under costal rim. (**b**) At age 14 (BMI = 15.2 kg/m^2^; −2.74 SD), the abdominal circumference increased by 9 cm, lower liver edge 12 cm under costal rim, lower spleen below the iliac spine (images from authors’ personal archive).

**Figure 4:**
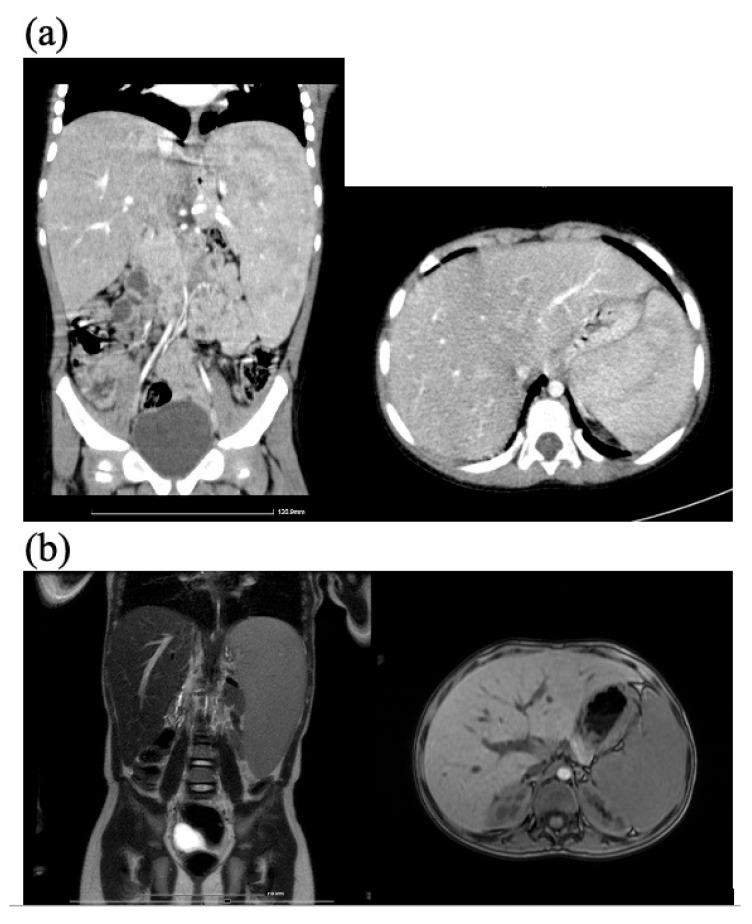
Hepatic and splenic volume evolution in NPD-A/B male: (**a**) abdominal CT scan at 4 years 7 months—liver volume 737.7 cm^3^, spleen volume 339.13 cm^3^; (**b**) abdominal MRI scan at 6 years 5 months—liver volume 892.66 cm^3^, spleen 486.11 cm^3^ (images from author’s personal archive).

Figure 3 depicts the clinical and Figure 4 the imagistic natural course of ASMD in two male patients diagnosed by the authors (images from personal archive).

Respiratory complications may occur in both types of Niemann–Pick Disease, type B being the most common. In ASMD type A, pulmonary involvement is rare, and if it emerges, it mainly consists of recurrent respiratory infections, interstitial lung disease, or aspiration pneumonia. Death in children with NPD-A typically happens due to respiratory failure by the age of 3 [14,32,34,36]. NPD-B patients exhibit specific pulmonary symptoms, such as interstitial lung disease (ILD), pulmonary hypertension, alveolar hypoventilation, upper airway obstruction (with or without obstructive sleep apnea syndrome), and recurrent airway infections caused by mucosal membrane swelling. Infants with ASMD type B may develop progressive respiratory symptoms and experience frequent respiratory infections due to aspiration, which can lead to severe respiratory failure, one of the main early causes of death in NPD-B patients [2,12,36,37,38]. The lung pathogenic process in ASMD involves accumulation of Niemann–Pick cells (“foam cells”) in the alveolar septum, bronchial walls, and pleura, causing a restrictive pattern. Histological examination shows intense blue staining with the May Grunwald–Giemsa and the Schmorl reaction—“sea-blue histiocytes”. These cells are large, multivacuolated and contain fine and coarse granules. Bronchoscopy detects the presence of Niemann–Pick cells in the bronchoalveolar lavage fluid and lung biopsy specimens [2,4,18,38].

**Figure 5:**
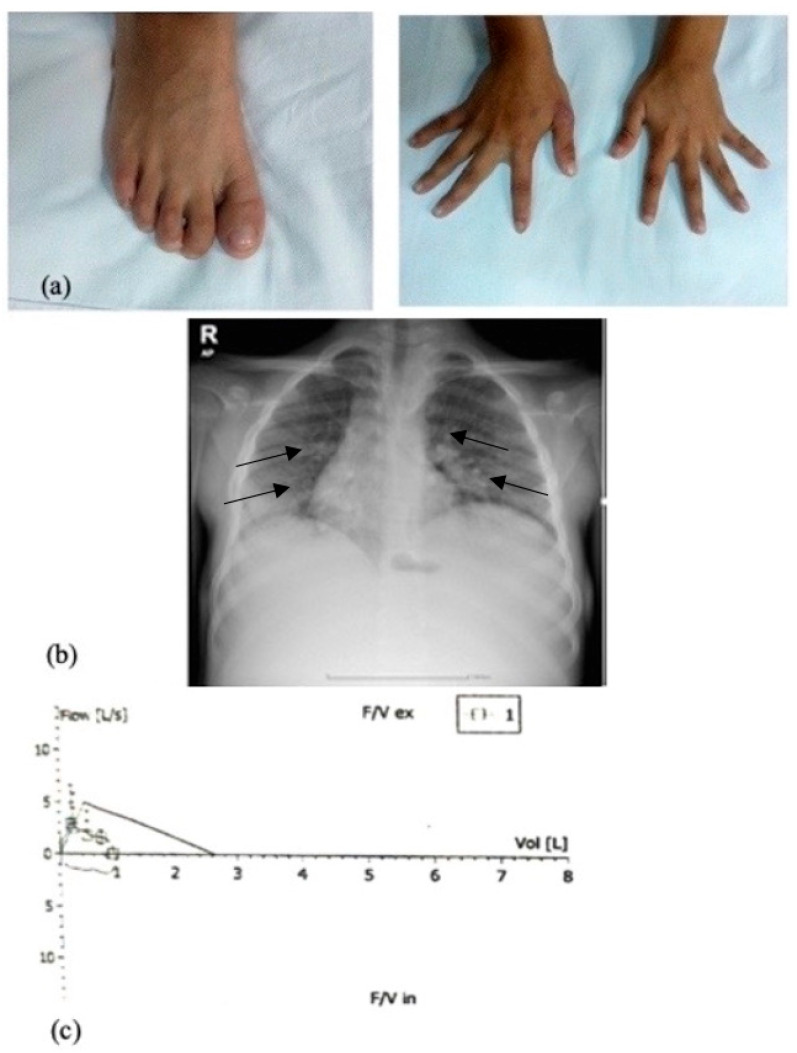
Respiratory findings in a 9 years old NPD-A/B male with (**a**) nail clubbing; (**b**) interstitial lung disease on thoracic X-ray; (**c**) pulmonary interstitial disease with reduced DL_CO_ (images from author’s personal archive).

The clinical clue to chronic pulmonary disease is the presence of clubbing fingers and toes (Figure 5a). X-rays may serve as a diagnostic tool in NPD type B, describing micronodular interstitial infiltrate. HRCT scans reveal basal interstitial lung disease (ILD) in most of the cases, with a thickened interlobular septum, which are primarily seen in lower lung zones and ground-glass opacities (Figure 5b), which are described often in the upper lung regions because alveoli are filled with Niemann–Pick cells. Pulmonary function tests usually show normal lung volumes with reduced diffusion capacity for carbon monoxide (DL_CO_) (Figure 5c) [4,32,38].

Echocardiography and catheter angiography are useful in NPD-B patients because they develop pulmonary hypertension with multiple arterio-venous fistulas [38].

**Figure 6:**
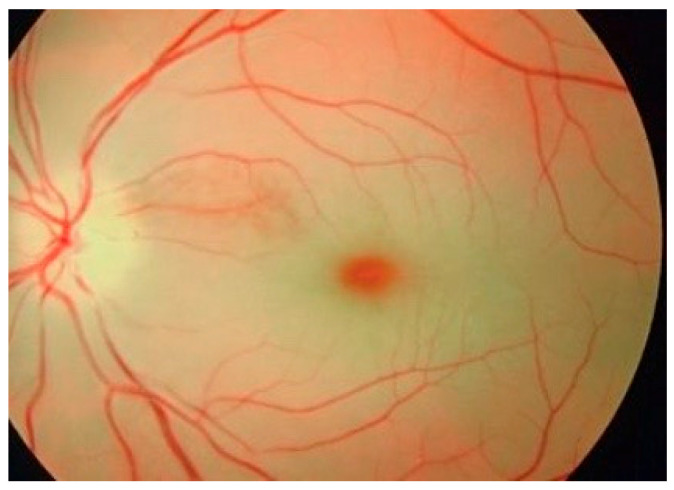
Red cherry macular spot on eye fundus examination [39].

Eye examination is important in ASMD patients. “Red cherry spot” refers to a red-tinted region in the center of the macula surrounded by retinal opacification. The peri-macular tissue of the retina becomes less transparent, while the fovea maintains its normal color, thus resulting in a red cherry spot appearance. In type A, the macular red cherry spot (Figure 6) is present in half of children at diagnosis moment, and by one year of age, all of them will exhibit this trait. On the other hand, only 1/3 of NPD-B patients display this sign [1,18,39].

NPD-A always presents with neurological manifestations such as seizures, hypotonia, and loss of muscle reflexes, while NPD-B patients have normal neurological development. Typical findings in brain imaging are cerebral atrophy or leukodystrophy, but usually scans come out negative. This, however, does not rule out later neurological impairment due to disease heterogeneity [2,4,5,18,31].

In July 2022, the Food and Drug Administration (FDA) approved a new and revolutionary drug for pediatric and adult populations with non-central nervous system ASMD, called Olipudase-alfa. This molecule is a recombinant human acid sphingomyelinase that reduces lipid build-up in organs and tissues of ASMD-affected patients and is intended as a lifelong enzyme replacement treatment (ERT) [8,40,41]. Efficacy of Olipudase-alfa was determined in three clinical studies: the ASCEND study for the adult population—a multicenter, randomized, double-blind, placebo-controlled phase II/III study [11]; ASCEND-Peds, which included young children and adolescents—a multicentric, open, phase I/II study [11,40,42]; and an extension study for both children over 5 years and adults [11].

Olipudase-alfa comes in 20 mg powder vials for intravenous infusion [11]. Patients receive progressively increased dosages via infusion pump every two weeks, until reaching a maintenance dose. Liver enzymes must be monitored throughout dosage escalation [11,40,41,42]. Treatment with Olipudase-alfa consists of two phases: first, a dosage escalation phase, which patients must undergo for at least 16 weeks in the pediatric population, and a maintenance phase, when the dosage has reached 3 mg/kg (Table 2) [11,41]. This type of administration is required because rapid, massive degeneration of sphingomyelin by ERT leads to large amounts of waste products with proinflammatory effects that can cause severe, immediate adverse reactions or increases in liver enzymes [11,41].

Olipudase-alfa has proved its efficacy in lung function. In the ASCEND-Peds open label trial, at week 52 compared with week 26 of treatment, there was a 33% mean increase in DL_CO_ from baseline (*p* = 0.0053) [42]. Regarding spleen and liver measurements, at 52 weeks compared to 26 weeks of Olipudase-alfa infusion, Diaz et al. observed a 49.2% mean decrease in spleen volume from baseline (*p* < 0.0001) and a 40.6% mean decrease in liver volume from baseline (*p* < 0.0001) [42]. ERT also exhibited great outcomes regarding height growth in children with ASMD. Mean height Z-scores improved from baseline after 1 year: +0.56, *p* < 0.0001 (N = 20) [9,40,42].

All outcomes showed further improvement with Olipudase-alfa treatment after 2 years (in ASCEND and ASCEND-Peds/LTS, also for lipid profile, liver enzymes, and platelet counts) or 6.5 years (in Phase 1b/LTS, also for lipid profile, liver enzymes). Olipudase-alfa continued to be well tolerated, and there were no new safety signals [8,9,10,21,35,40,41,42,43].

Solid organ transplantation is another open treatment possibility for NPD patients. However, getting rid of a damaged organ does not prevent recurrence of disease in the transplanted one, nor progression of disease in other organs or tissues [38,44,45,46,47]. Lung transplantation should be considered in NPD-B patients with severe respiratory impairment [38,45,46]. Liver transplantation might be a viable therapeutic option for both ASMD type A and B, keeping in mind that hepatic failure is one of the leading causes of death in Niemann–Pick disease. Organ replacement should be performed when the liver becomes cirrhotic and complications emerge, such as portal hypertension, ascites, esophageal varices, and hepatic encephalopathy [38,44,47]. Bone marrow transplantation may alleviate hepato-splenomegaly and improve blood count, but possible complications (graft rejection, renal failure, etc.) make this therapy option highly unrecommended [38].

The ultimate treatment-step is gene therapy, though its efficacy and safety have not yet been demonstrated in humans [38].

## 2. Review of the Published ASMD Cases in the Literature and Case Data from the Authors’ Personal Experience

Published literature is scarce regarding ASMD cases, so the authors aimed to add to the existing data a comprehensive review of the data published in the last 10 years and details from their personal experience with the diagnosis of two patients with NPD-A/B and their enzyme replacement treatment.

This literature review is based on PubMed papers published in the last 10 years (from 2014 to January 2025) regarding Niemann–Pick disease type A and B characteristics, from genetic status to therapy options, with a primary interest in the pediatric field. The following particular search terms in different combinations with filters for case reports, clinical trials, and reviewed articles were used in order to find the most suitable papers for this review: “Niemann–Pick disease”, “acid-sphingomyelinase deficiency”, “genetic traits in NPD-A and NPD-B”, “enzyme replacement therapy”, “olipudase-alfa”, “metabolic disorder” or “sphingomyelin”. Inclusion criteria were a definite diagnosis of Niemann–Pick disease type A, B, or A/B; confirmation of diagnosis by biopsy, enzyme activity level, or molecular assay; and proper evaluation of main affected organs/tissues (liver, spleen, lungs, central nervous system). Exclusion criteria were non-ASMD lysosomal storage disorders, incomplete patient history or diagnostic work-up, non-English published papers, and adult patients with NPD-A, B, A/B, or NPD-C.

All papers concerning Niemann–Pick disease type A and B in children were reviewed (which were published since 2014). A total of 26 of the 62 publications cited in this paper included data regarding children, whether they were case reports, series of cases, or enzyme replacement therapy outcomes. Two papers detailed case reports describing children with NPD type C. One publication outlined a prospective study regarding causes of death in chronic visceral and chronic neurovisceral ASMD but included both children and adults. In the 19 publications detailing case reports (16) and series of cases (3), we assessed number of patients, gender distribution, type of ASMD, symptoms and diagnosis onset age, organ involvement, type of diagnosis, gene mutation, vital status, enzyme replacement therapy, and its outcome.

According to the current reviewed literature, acid sphingomyelinase deficiency types A and B are a rare disease among the pediatric population; from 2014 to January 2025 we identified 30 cases. Table 3 comprises all ASMD cases published in the last decade.

The male/female ratio was 1.4 (one case without gender information). The youngest patient at disease onset was a female newborn with NPD-A. The lowest diagnosis age was 4 months. The longest timeframe between onset symptoms and diagnostic moment was 5 years and 3 months (onset age at 9 months to diagnosis at 6 years); it was a case of chronic visceral non-CNS ASMD.

Sphingomyelin and other lipids accumulate in hepatocytes, monocytes, and macrophages, which leads to organ damage and dysfunction. Therefore, liver and spleen functions and volumes should become part of routine assessment of NPD children, in order to observe disease progression [2,4]. McGovern et al. state that in children, the mean liver volume was 2.2 ± 0.7 MN at baseline, 2.1 ± 0.7 at 1 year, and 1.7 ± 0.4 at the final visit [31]. Moderate to severe hepatomegaly was observed in 96% at baseline, 97% at 1 year, and 88% at the final visit [31]. McGovern et al. concluded that individuals with significant splenomegaly were at higher chance of death than those with smaller or intact spleens (odds ratio = 10.29, 95% CI: 1.7, 62.7) [31].

Both our NPD patients exhibited enlarged liver and spleen, with dimensions progressively increasing over the years. The older NPD-A/B patient had massive hepatomegaly and splenomegaly, starting from an abdominal perimeter of 70 cm at 9 years of age, with lower liver edge at 10 cm and lower spleen edge at 9 cm under the costal rib; by age 14, the abdominal perimeter went up 9 cm (Figure 3)**.** This patient’s CT scan showed massive organomegaly, with a spleen volume of 2201 cm^3^ and liver volume of 2213 cm^3^. Spleen and liver volumes significantly increased over the course of two years in the younger NPD-A/B boy as well, the liver volume from 737.7 cm^3^ to 892.66 cm^3^ and spleen volume from 339.13 cm^3^ to 486.11 cm^3^ (Figure 4).

Laboratory findings in both types of ASMD may include lipid abnormalities such as reduced HDL cholesterol, hypertriglyceridemia, and elevated LDL cholesterol (which contributes to cardiac disease), also liver cytolysis and cholestasis [18,32,38,48,49,50,51]. Kavcic et al. reported on NPD-A in an 11-month-old boy with increased total cholesterol (5.2 mmol/L), decreased HDL-cholesterol (0.6 mmol/L), and high LDL and triglyceride levels [34]. Velarde-Felix et al. reported on a 16-year-old Mexican girl with NPD-B and persistently low levels of HLD-cholesterol [52].

Platelet count must also be included, especially in NPD-B patients who exhibit frequent mild to severe thrombocytopenia and subsequent bleeding [12,28]. Ceron-Rodriguez et al. reported on a 6-year-old girl with NPD-B epistaxis episodes occurring once a month on average [53], and Sideris et al. found easy bruising or nasal bleeding in an 8-year-old girl with NPD-B [37]. Regarding the 19 publications reviewed, all patients, whether they were diagnosed with NPD-A or NPD-B, displayed hepatic and spleen involvement, the marked enlargement of the liver and spleen, and altered laboratory findings (cytolysis, dyslipidemia, low platelet number) [13,14,18,19,21,22,32,34,35,37,43,52,53,54,55,56,57,58,59]. This is also true for our 14-year-old patient with NPD-A/B. Despite significant hepatosplenomegaly as presented, the 6-year-old NPD-A/B patient had normal liver enzymes and hematologic counts.

**Table 3:** Literature cases published on ASMD types A, B, and A/B (2014–2024).

Article by Type and Year	Subject Age/Gender	Type of ASMD	Onset Age	Diagnosis Age	Organ Involvement	Type of Diagnosis	Gene Involved	Vital Status	ERT	ERT Outcome
Sideris et al., 2016 [37] Case report	8 years Female	NPD-B	9 month	6 years	Lungs Liver Spleen	Genetic	Homozygous c.C947A	Alive	NO	/
Van Baelen A. et al., 2024 [35] Case report	1 yr 10 month Male	NPD-B	7 month	3 years	Liver Spleen	Enzyme activity Genetic	Homozygous (deletion) c.1829_1831	Alive	Since 4 years 7 month; 16 weeks dose escalation	No AR No hepatomegaly Mild splenomegaly Growth failure recovered No heart/lung involvement
Ngoenmak T. et al., 2023 [32] Case report	7 month Female	NPD-A	2 month	7 month	Lungs Liver Spleen Red cherry spot Developmental delay	Liver biopsy Enzyme activity Genetic	Homozygous c.1214T>C	Died at 4 years (respiratory failure and neurological deterioration)	NO	/
Ota S. et al., 2020 [14] Case report	4 month Female	NPD-A	4 month	4 month	Lungs Liver Spleen Red cherry spot Developmental delay	Enzyme activity Genetic	p.C133Y	Died at 3 years 1 month (liver and respiratory failure)	NO	/
Gul F. et al., 2024 [13] Case report	11 month Male	NPD-A	5 month	11 month	Lungs Liver Spleen Growth failure Developmental delay Hypotonia	Liver biopsy NO genetic NO enzyme activity	/	Alive (1 yr 7 month)	NO	/
Kavcic A. et al., 2022 [34] Case report	11 month Male	NPD-A	3 month	10 month	Lungs Liver Spleen Growth failure Developmental delay Hypotonia	Liver biopsy Enzyme activity Genetic	Compound Heterozygous (both pathogenic variants) C573delT (from monthther) c.1267C>T (from father)	Alive	NO	/
Velez Pinos P.J. et al., 2022 [18] Case report	4 years 3 month Male	NPD-A/B	2 month	4 years 3 month	Lungs Liver Spleen Growth failure NO neurological findings	Live biopsy Bone marrow biopsy Enzyme activity Genetic	Compound Heterozygous (both pathogenic variants) c.28C>7 c.362T>C	Alive	NO	/
Dalal P.G. et al., 2024 [54] Case report	1 yr 2 month Gender unknown	NPD-A	/	/	Liver Spleen Growth failure Developmental delay Hypotonia	Enzyme activity Genetic	Compound Heterozygous (both pathogenic variants) c.573delT c.1783_1784delCT	Alive	NO	/
Mirani E. et al., 2021 [55] Case report	1 yr 6 month Male	NPD-A	6–7 month	1 yr 6 month	Liver Spleen Lungs Growth delay Developmental delay	Bone marrow biopsy Enzyme activity	/	Alive	NO	/
Deodato F. et al., 2024 [43] Case report	8 month Male	NPD-A/B	4 month	6 month	Liver Spleen Red cherry spot NO neurological findings	Enzyme activity Genetic	Homozygous c.739G>A	Alive	Since 8 month; 16 weeks dose escalation	Transient IgG-AMA AR: fever Improved lipid profile AST, ALT normalized Growth failure recovered Height from P25 to P75 Developed neurological impairment at 22 month
Hashemian S. et al., 2019 [56] Prospective study 2012->2016	Male	NPD-A	/	6 month	Liver Spleen Red cherry spot Developmental delay	Genetic	c.740delG	Died at 6 month	NO	/
	Male	NPD-A	/	5 month	Liver Spleen Developmental delay	Genetic	c.740delG	Died at 2 years 6 month	NO	/
	Female	NPD-B	/	6 month	Liver Spleen Developmental delay	Genetic	c.108delG	Died at 3 years	NO	/
	Male	NPD-B	/	6 month	Liver Spleen Developmental delay	Genetic	/	Died at 4 years 6 month	NO	/
	Female	NPD-B	/	6 month	Liver Spleen Developmental delay	Genetic	c.1110delT	Died at 3 years	NO	/
	Male	NPD-B	/	7 month	Liver Spleen Developmental delay	Genetic	c.573delT	Died at 1 yr 6 month	NO	/
	1 yr 6 month Male	NPD-B	/	/	Liver Spleen Red cherry spot Developmental delay	Genetic	c.1390G>T	Alive	NO	/
	2 years 4 month Female	NPD-B	/	/	Liver Spleen Developmental delay	Genetic	c.1524G>A	Alive	NO	/
Pan Y.W. et al., 2023 [21] Case series	Male	NPD-A/B	< 3 years	3 years 2 month	Liver Spleen Lungs Red cherry spot Growth failure Developmental delay	Genetic	Compound Heterozygous c.1486+5G>C (from father) c.1497_1498delGTinsAC (from monthther)	Alive	Since 5 years 8 month	No severe AR IgG-AMA Reduced liver and spleen volume Lipid profile normalized Improved lung function (improved “ground-glass” aspect) WBC number improved Weight improved Height still under limit
	Male	NPD-A/B	< 1 yr	1 yr 11 month	Liver Spleen Lungs Red cherry spot Growth failure Developmental delay	Genetic	Compound Heterozygous c.1486+5G>C (from father) c.1498T>C (from monthther)	Alive	Since 2 years 6 month	No severe AR Transient elevation AST, ALT Reduced liver and spleen volume Lipid profile normalized Improved lung function (improved “ground-glass” aspect) WBC number improved Weight improved Height still under limit
Tangde A. et al., 2017 [19] Case report	1 yr 6 month Male	NPD-A	2 month	1 yr 6 month	Lungs Liver Spleen Developmental delay	Bone marrow biopsy Spleen aspiration Liver biopsy	/	?	NO	/
Taha I. et al., 2023 [22] Case report	13 years Male	NPD-A/B	/	13 years	Liver Spleen NO neurological findings	Enzyme activity Genetic	Heterozygous c.739G>A c.1829_1831del	Alive	Awaiting ERT	/
Aghamandi F. et al., 2022 [57] Case report	1 yr Male	NPD-A	9 month	1 yr	Liver Spleen Red cherry spot Developmental delay Hypotonia Seizures	Enzyme activity Genetic	Homozygous c.682T>G	Alive	NO	/
Sunil Mohan M. et al., 2014 [58] Case report	11 month Female	NPD-A	/	11 month	Liver Spleen Lungs Growth failure Developmental delay	Bone marrow biopsy Enzyme activity	/	Died at 1 yr 1 month (respiratory failure)	NO	/
Ceron-Rodriguez M. et al., 2018 [53] Case series	1 yr 3 month Female	NPD-A	neonatal	1 yr 3 month	Liver Spleen Lungs Growth failure Developmental delay	Enzyme activity Genetic	Homozygous c.1343A>G	Died at 1 yr 5 month (pneumonia)	NO	/
	5 years Female	NPD-B	3 years	5 years	Liver Spleen Growth failure	Enzyme activity Genetic	Compound Heterozygous c.1343A>G c.1829_1831delGCC	Alive	NO	/
	7 years Female	NPD-B	2 years	7 years	Liver Spleen Lungs Growth failure	Liver biopsy Enzyme activity Genetic	Compound Heterozygous c.1547A>G c.1805G<A	Alive	NO	/
	6 years Female	NPD-B	2 years	6 years	Liver Spleen	Enzyme activity Genetic	Homozygous c.1263+8C>T	Alive	NO	/
Velarde-Felix J.S. et al., 2016 [52] Case report	16 years Female	NPD-B	/	/	Liver Spleen NO neurological findings	Bone marrow biopsy Genetic	Heterozygous (missense) c.1343A>G c.1426C>7			
Shubhankar M. et al., 2014 [59] Case report	9 month Male	NPD-A	6 month	9 month	Liver Spleen Red cherry sport	Liver biopsy	/	?	NO	/

“NO”—patient has not received enzyme replacement treatment. “?”—patients status (alive or dead) is unknown.

The literature data describe macular red cherry spots in all children with NPD-A by the age of 1 year and only in 1/3 of those with NPD-B [18]. Nine patients out of thirty showed this sign upon fundus eye examination (five of them were diagnosed with NPD-A, three with NPD-A/B, and only one had NPD-B). In their 5-year prospective study, Hashemian et al. reported only eight ASMD patients; two of them exhibited red cherry spots, a 6-month-old boy with NPD-A and 1.5-year-old male with NPD-B [56]. Both our patients exhibited this ophthalmologic sign.

The pulmonary pathogenic process in NPD involves the accumulation of “foam cells” in the alveolar septum, bronchial walls, and pleura, causing a restrictive pattern [2,4,18]. In NPD-A, lung involvement consists of recurrent respiratory infections that constitute the main cause of death (respiratory failure) by the age of three [14,32,34,36]. NPD-B patients primarily exhibit interstitial lung disease [2,38], as did our older patient since the age of 5. A total of 13 children exhibited associated lung disease (8 with NPD-A, 2 with NPD-B, 3 with NPD-A/B). A total of four out of eight patients with ASMD type A and pulmonary disease died due to respiratory complications. Those with chronic visceral and chronic neurovisceral ASMD are still alive, despite pulmonary issues such as dependency on supplemental oxygen and recurrent chest infections.

A total of 11 children exhibited associated growth impairment: 6 with NPD-A, 3 with NPD-A/B, and 2 with NPD-B. Gul et al. reported on an 11-month-old boy with NPD-A at 68 cm in height and 5.3 kg, both below the 3rd percentile in WHO growth charts [13]. Ceron-Rodriguez et al. outlined two cases of NPD-B (5 years and 7 years, both females) with stature and body weight below the 3rd percentile for their age [53]. Both our patients have low stature, below the 3rd percentile for their age.

NPD-A can be distinguished by NPD-B by measuring low ASM activity in designated cells [29]. In 15 cases, enzyme levels were used as a diagnostic tool, but never alone; genetic assay and liver/spleen/bone marrow biopsy were associated in order to establish an unquestionable diagnosis.

**Figure 7:**
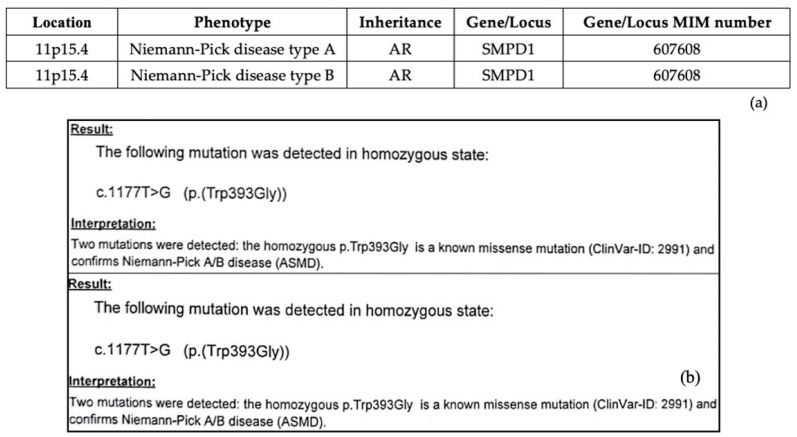
(**a**) Genotype–Phenotype relationship of NPD-A and NPD-B in the OMIM system [7]; (**b**) genotypes of the authors’ cases: NPD-A/B.

Genetic testing for pathogenic mutations remains the gold-standard diagnostic tool for NPD [15,17]. ASM deficiency has been defined in the Online Mendelian Inheritance in Men (OMIM) database [7]. Both NPD-A and NPD-B are caused by a homozygous or compound heterozygous mutation in the SPMD1 gene on the chromosome 11p15 (Figure 7a) [15,16]. Genotyping in the authors’ two patients revealed a homozygous state for (p.(Trp393Gly)), a known missense mutation that confirmed NPD type A/B (Figure 7b).

The SMPD1 gene encodes an enzyme called acid sphingomyelinase, which breaks down sphingomyelin into ceramide and phosphocholine [16]. Type A is always inherited in an autosomal recessive fashion and is marked by small deletions or nonsense mutations that produce a cut-short version of ASM peptides, but also missense mutations that generate a non-catalytic enzyme [38]. Type B is not always an autosomal recessive disorder, although this is the main mode of inheritance. Some individuals are carriers, which means they are heterozygous for the mutation but can still present with milder forms of disease. NPD-B is mainly caused by missense mutations that result in a residual enzymatic catalytic activity [17,38].

More than 100 mutations in the SMPD1 gene are cited in the Human Gene Mutation Database (HGMD) for ASMD type A and B [16]. Over 60% are missense mutations, and 19% are frameshift mutations [38]. R496L, L302P, and fsP330 represent over 90% of the Ashkenazi mutant alleles and are linked to ASMD type A [17]. The most frequent mutation reported worldwide is a deletion associated with a mild form of NPD-B disease [38]. “Neuroprotective” status results from residual activity of ASM due to a common mutation of the SMPD1 gene found in type B patients, R608 [17].

In 25 out of 30 cases, a genetic assay was performed (15 were homozygous; in Hashemian et al.’s study, one child with NPD-B had no information regarding genetic status; nine were heterozygous [56]). Gul et al. established NPD-A diagnosis in an 11-month-old boy only on the basis of liver biopsy [13]. Mirani et al. proved NPD-A in a 1.5-year-old male by measuring ASM activity and performing a bone marrow biopsy [55]. Tangde et al. indicated NPD-A in a 1-year-6-month-old boy on the basis of liver, spleen, and bone marrow biopsies [19]. Sunil Mohan et al. described NPD-A in an 11-month-old girl by measuring ASM levels and performing bone marrow aspiration [58]. Shubhankar et al. established an NPD-A diagnosis in a 9-month-old boy only after liver biopsy [59]. All five cases without genetic assessment have one thing in common: they are all cases of ASMD type A, which embraces a high index of clinical suspicion in an infant with developmental delay, growth impairment, and hepatosplenomegaly. All this, alongside a low enzymatic activity and the presence of “foamy cells” at microscopy, leads to NPD-A diagnosis.

The Department of Health and Human Services in the U.S. developed a list called “The recommended universal screening panel”, or RUSP, which identifies various genetic disorders, but Niemann–Pick disease is not included [33]. Currently in America, only two States—Illinois and New Jersey—offer newborn screening for ASMD [33]. Geberhiwot et al. elaborated two consensus clinical management guidelines for NPD-A, NPD-B, NPD-A/B, and NPD-C, papers published in the Orphanet Journal of Rare Diseases back in 2018 and 2023, which clearly state the steps of correct diagnosis for these types of ASMD [27,30]. As for Romania, a statement paper was recently published in the Romanian Journal of Pediatrics presenting a screening program developed in the Department of Pediatrics of “Grigore Alexandrescu” Emergency Children’s Hospital, Bucharest, for those patients with a high index of clinical suspicion [60].

Since Olipudase-alfa was approved for pediatric and adult populations with non-central nervous system ASMD, ERT via intravenous infusion every two weeks has emerged as a treatment. Dosages are progressively scaled-up for at least 14 weeks (for children) and 16 weeks (for adults), until a maintenance dose is reached (3 milligrams per body kilogram) [8,11,41].

Four children received Olipudase-alfa (one with NPD-B, three with NPD-A/B) [21,35,43]. A 13-year-old boy with NPD-A/B was awaiting therapy back in 2023 (Taha et al.) [20]. Van Baelen et al. reported on NPD-B in a boy, diagnosed at 3 years of age, who started ERT at 4 years 7 months of age [35]. Deodato et al. presented NPD-A/B in a boy, diagnosed at 6 months of age, who began ERT two months after diagnosis [43]. Pan Y.W. et al. described two cases of NPD-A/B in boys diagnosed at 3 years 2 months and 1 year 11 months, respectively, who were treated starting at 5 years 8 months and 2.5 years, respectively [21]. In two cases, Olipudase-alfa dosages were escalated over a period of 16 weeks.

**Figure 8:**
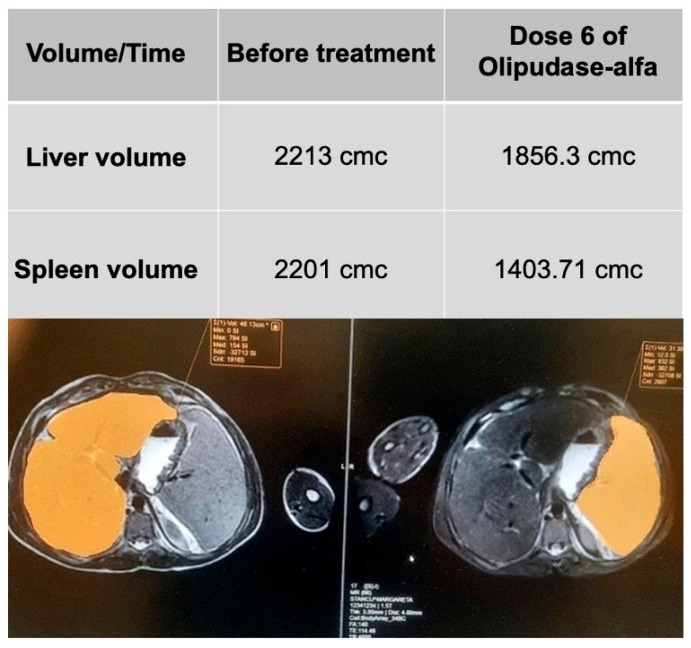
Evolution of liver and spleen volumes in NPD-A/B male after six doses of Olipudase-alfa (images from authors’ personal archive).

The authors included two patients in the treatment protocol in the last year (a 15-year-old male with NPD-A/B and a 6-year-old male with NPD-A/B). Figure 8 exhibits the evolution of liver and spleen volumes after six doses of Olipudase-alfa in the 15-year-old male with NPD-A/B. No side effects were noted so far.

Regarding intravenous-infusion-like adverse reactions, the pediatric population takes the leading spot. Children may exhibit mild symptoms from fever, nausea, vomiting, or headaches to a severe phenomenon like anaphylaxis. The main medication-like adverse reaction is an increase in liver enzymes, which is why ASMD disease monitoring requires routine check-ups of transaminase levels. After a few weeks of replacement therapy, anti-medication antibodies (AMAs) may emerge [8,11,41,61]. In the ASCEND-Peds study, 65% of children became AMA positive, but only five of them maintained that immunological status [11].

Van Baelen et al. reported no adverse reactions (ARs), no liver enlargement, and mild splenomegaly. Growth failure recovered, and no heart or lung involvement was described [35]. Deodato et al. reported fever after ERT infusions and transient anti-medication antibodies (AMAs). In this case, the lipid profile improved, and liver cytolysis disappeared. The child recovered his growth status, with his height reaching the 75th percentile (at the beginning, his stature was in the 25th percentile). Unfortunately, he developed neurological impairment after 14 months of ERT [43]. Pan Y.W. et al. reported two children who received ERT; at first, they did not exhibit severe ARs but developed IgG-AMA; liver and spleen volume decreased, lipid profile normalized, lung function improved, WBC number enhanced, body weight picked-up, but stature stayed below the limit-line [21]. The second child had a transient elevation of liver enzymes, but liver and spleen volume went down; the lipid profile normalized, the pulmonary “ground-glass” pattern improved, body weight went up, but stature was not exceeded [21].

## 3. Conclusions

ASMD presents with significant variability in its clinical manifestation, ranging from the severe, early-onset neurodegeneration seen in NPD-A to the more slowly progressive visceral involvement without neurological deterioration in NPD-B. The intermediate NPD-A/B phenotype shows a slower progression, with both visceral and neurological features. The diversity in age of onset, organ involvement, and disease severity highlights the need for precise genetic testing and tailored clinical management for each. Early diagnosis and understanding of the subtype are crucial for proper prognostic counseling and treatment planning.

The literature is scarce on this subject, thus making the discovery of new cases harder. However, ASMD should be takin into consideration when confronted with a child with liver and spleen enlargement. A rapid laboratory assessment of WBC and platelet number alongside a basic abdominal ultrasound should provide sufficient information to raise clinical suspicion of the disease. Given the complex nature of ASMD, early and accurate diagnosis is essential for providing appropriate medical care. This involves not only genetic and enzymatic testing but also regular monitoring of organ function (e.g., liver, spleen, and lungs) and neurological status. A multidisciplinary approach is vital, including genetic counseling, symptom management, and consideration of advanced treatments like ERT. Moreover, new therapies such as gene therapy and organ transplantation are still being explored, emphasizing the importance of ongoing research and clinical trials to address the limitations of current treatments.

Olipudase-alfa is showing promising results in treating non-neurological forms of ASMD. It has been particularly effective in reducing organomegaly (liver and spleen volumes), improving lung function, and promoting growth in pediatric patients. ERT represents a significant advancement in managing the visceral symptoms of NPD-B and the intermediate NPD-A/B phenotype. However, it is important to note that ERT has not been proven effective for neurological involvement, which remains a challenge for patients with NPD-A and some with NPD-A/B.

In conclusion, while significant progress has been made in the understanding and treatment of ASMD, ongoing research, early diagnosis, and personalized care remain essential to improving outcomes and quality of life of affected individuals. And as with any rare disease, each new case provides the medical community with valuable information needed to achieve these goals.

## Figures and Tables

**Table 1:** Main features of NPD type A, B, and A/B [1,4,5,20].

	NPD Type A	NPD Type B	NPD Type A/B
Onset	Early infancy	Any time in life	Infancy to childhood
Signs and Symptoms	Hepatosplenomegaly Jaundice Red cherry macular spot Loss of reflexes, muscle tone Feeding difficulties Growth delay Rapid neurodegeneration Interstitial lung disease Atherogenic lipid profile	Splenomegaly Liver enlargement Red cherry macular spot Slow neurodegeneration Growth delay Interstitial lung disease Thrombocytopenia Anemia Low white blood cells Atherogenic lipid profile	Slower progression Variable multiorgan manifestations Neurodegeneration
Severity	Life-threatening	Slowly progressive	Slow neurologic degeneration
Life expectancy	2 to 3 years	Childhood to late adulthood	Childhood to mid-adulthood

**Table 2:** Olipudase-alfa dosage escalation protocol in pediatric population [11].

	Dosage Escalation								Maintanance Dosing
Start	Week 2	Week 4	Week 6	Week 8	Week 10	Week 12	Week 14	Week 16	Every 2 Weeks
0.03 mg/kg	0.1 mg/kg	0.3 mg/kg	0.3 mg/kg	0.6 mg/kg	0.6 mg/kg	1.0 mg/kg	2.0 mg/kg	3.0 mg/kg	3.0 mg/kg

## Data Availability

Data are contained within the article.
